# Karyotype variability in six Amazonian species of the family Curimatidae (Characiformes) revealed by repetitive sequence mapping

**DOI:** 10.1590/1678-4685-GMB-2021-0125

**Published:** 2022-06-27

**Authors:** Juliana Nascimento Moraes, Patrik Ferreira Viana, Ramon Marin Favarato, Vanessa Susan Pinheiro-Figliuolo, Eliana Feldberg

**Affiliations:** 1 Instituto Nacional de Pesquisas da Amazônia (INPA), Coordenação de Biodiversidade, Laboratório de Genética Animal, Manaus, AM, Brazil.

**Keywords:** Cytogenetics, rDNA, ITS, chromosomal rearrangements

## Abstract

Fishes of the Curimatidae family represent one of the most important freshwater ichthyofauna groups of Central and South America, with 117 recognized species distributed in eight genera. In this study, six species - *Curimata inornata*, *Curimatella dorsalis*, and *Psectrogaster falcata* collected from the Lower Araguaia River, Pará, Brazil; *Curimata vittata*, *Curimatella meyeri*, and *Psectrogaster rutiloides* collected from the Catalão Lake, Amazonas, Brazil - were cytogenetically analyzed, investigate the occurrence and distribution of repetitive DNA classes in the karyotypes. All species had 2n=54 metacentric/submetacentric chromosomes. Despite the conservative diploid number, we observed variations in the karyotypic structure among species. Ribosomal DNA (rDNA) 18S and 5S were found in single or multiple sites, with the first report of synteny in *Curimatella dorsalis*, and the occurrence of several interstitial telomeric sequences (ITSs) in species of the genera *Curimatella* and *Psectrogaster*. Interspecific karyotypic diversity both concerning structure and location/position of the nucleolar organizer regions (NOR) and ribosomal DNA, suggesting the occurrence of several non-Robertsonian rearrangements driving the evolution of this family.

The Curimatidae family currently encompasses 117 fish species, alocated in eight genera: *Curimata*, *Curimatella*, *Curimatopsis*, *Cyphocharax*, *Potamorhina*, *Psectrogaster*, *Pseudocurimata*, and *Steindachnerina* (Fricke et al., 2021). The species are widely distributed throughout Central and South America River basins, inhabiting different aquatic environments. Ecologically, these fishes have an important role as food resources for larger predatory fish and act in recycling organic material due to detritivores’ eating habits, being easily distinguished from the other taxa of the Characiformes order by their complete absence of teeth (Vari, 1989, 2003).

Cytogenetically, this family shows 2n=54 with biarmed chromosomes as the most frequent in the analyzed species (Table 1). However, despite this apparent conservative karyotype and chromosome morphology, variations in diploid number have been reported in at least six species, in addition to the occurrence of B chromosomes, as well as interspecific variation in the location/position of the nucleolar organizer regions (NORs) (Venere and Galetti, 1989; Feldberg et al., 1992; Navarrete and Júlio-Júnior, 1997; Brassesco et al., 2004; Venere *et al.*, 2008) (Table 1).

The chromosomal mapping of repetitive sequences, such as 5S and 18S ribosomal DNAs (rDNA) and telomeric DNA (TTAGGG)_n_, has proven to be an excellent tool for the chromosomal characterization in different groups of Neotropical fishes (Cioffi and Bertollo, 2012; Viana et al., 2017; Ferreira et al., 2020), providing a set of relevant information that can contribute to cytotaxonomy, elucidate geographic distribution patterns and evidence sex chromosomes. In Curimatidae, even with scarce data on mapping these sequences, evident interspecific differences were already observed (De Rosa et al., 2006, 2007; Teribele et al., 2008; Oliveira, 2010; Pinheiro et al., 2016; Sampaio et al., 2016) (Table 1).

**Table 1 -:** Overview of cytogenetic data of fish species from the Curimatidae family. 2n= diploid number; FN= fundamental number; Ag-NOR= nucleolar organizer region; m= metacentric; sm= submacentric; st= subtelocentric; a= acrocentric; B= supernumerary chromosome; p= short arm; q= long arm; t= terminal; i= interstitial; pc= pericentromeric; c= centromeric; ITS= interstitial telomeric sequence; -= nonexistent data.

Species	Locality	2n	FN	Karyotype formula	Ag-NOR pair / position	C Banding	rDNA 18S Pair / position	rDNA 5S Pair / position	Telomere	Reference
*Curimata*										
*C. cyprinoides*	Negro and Solimões River/AM	54	108	44m+10sm	3m/*q*t	-	-	-	-	3
	Araguaia River/MT	54	108	44m+10sm	7m/*q*t	-	-	-	-	16
*C. inornata*	Negro and Solimões River/AM	54	108	40m+14sm	21sm/*p*i					3
	Araguaia River/MT	54	108	40m+14sm	3m22sm/*q*t	pc/t	-	-	-	16
	Araguaia River/PA	54	108	38m+16sm	20sm/*p*t	pc/t	20sm/*p*t	9m/*p*i	t	22
*C. knerii*	Negro and Solimões River/AM	54	108	40m+12sm+2st	27st/*p*t	-	-	-	-	3
*C. ocellata*	Uatumã River/AM	56	112	40m+16sm	26sm/*p*i	-	-	-	-	3
*C. vittata*	Negro and Solimões River/AM	54	108	42m+12sm	sm/*q*t	-	-	-	-	3
	Catalão Lake/AM	54	108	38m+16sm	20sm21sm/*q*t	pc/t	20sm21sm/*q*t	25sm/*p*i	t	22
*Curimatella*										
*C. alburna*	Negro and Solimões River/AM	54	108	46m+8sm	14m/*q*t	-	-	-	-	3
*C. dorsalis*	Miranda River/MS	54	108	46m+8sm	13m/*p*t	pc	-	-	-	7
	Paraná River/AR	54	108	54m/sm	2m/*q*t	c/t	-	-	-	11
	Araguaia River/PA	54	108	44m+10sm	2m/*q*t	pc/t	2m/*q*t	2m/*q*i	t/ITS 18 pairs	22
*C. immaculata*	Araguaia River/GO	54	108	46m+8sm	24sm/*q*t	-	-	-	-	16
*C. lepidura*	São Francisco River/SP	54	108	54m/sm	9m/*p*t	-	-	-	-	2
*C. meyeri*	Negro and Solimões River/AM	54	108	46m+8sm	9m/*q*t	-	-	-	-	3
	Catalão Lake/AM	54	108	46m+8sm	9m/*q*t	pc/t/i	7m/*p*t 9m/*q*t	26sm/*p*i	t/ITS 14 pairs	22
*Curimatopsis*										
*C. myersi*	Miranda River/MS	46	92	42m+4sm	-	-	-	-	-	7
*Cyphocharax*										
*C. gilbert*	Paraibuna River/SP	54	108	44m+10sm	2m/*p*t	pc/t	-	-	-	16
*C. cf gilli*	Bento Gomes River/MT	54	108	54m/sm	1m/*q*i	-	-	-	-	2
*C. gouldingi*	Araguaia River/GO	54	108	54m+B	2m/*q*t	-	-	-	-	16
*C. modestus*	Águas de São Pedro/SP	54	108	54m/sm	2m/*q*t	-	-	-	-	2
	Três Bocas Stream/PR	54	108	54m/sm+B	2m/*q*t	pc/t	2/*q*t	-	-	6,13,15,19,20
	Mogi-Guaçu River/SP	54	108	54m/sm+B	-	pc	-	-	-	8
	Taquari River/PR	54	108	54m/sm+B	2m/*q*t	pc/t	2/*q*t	-	-	13,15
	Tibagi River/PR	54	108	54m/sm	2m/*q*t	-	2/*q*t	-	-	15
	Água da Floresta River/PR	54	108	54m/sm	2m/*q*t	-	2/*q*t	-	-	15
	Paranapanema River/SP	54	108	54m/sm+B	2m/*q*t	pc/t	2/*q*t	3,20/*p*i	-	12,14,17
	Tietê River/SP	54	108	54m/sm+B	2m/*q*t	pc/t	2/*q*t	3,20/*p*i		1,12,14,17
*C. naegelii*	Mogi-Guaçu River/SP	54	108	54m/sm	25/*p*t	-	-	-	-	2
	Mogi-Guaçu River/SP	54	108	46m+8 sm	1,2,11/*q*t 6/*pq*t 21/*p*t	pc/t	-	-	-	16
	Ribeirão Minhoca/MG	54	108	54m/sm+B	6/*q*t	pc/t	6/*q*t	3,20/*p*i	t/ITS 2 pairs	18
*C. platanus*	Paraná River/AR	58	116	52m/sm+6st	5m/*p*t	-	-	-	-	11
	Pirá-Pytá Stream/AR	58	116	48m+4sm+6st	6m/*p*t	pc/t	-	-	-	16
*C.* cf *spilurus*	Madeira River/RO	54	108	54m/sm	10m/*q*t	-	-	-	-	2
*C. spilotus*	Paraná River/AR	54	108	54m/sm+B	1/*q*i	pc/t	-	-	-	10,11
	Capivara Stream/RS	54	108	54m/sm+B	2/*q*t	pc/t	2/*q*t	-	-	19,20
	Gasômetro/RS	54	108	54m/sm+B	2/*q*t	pc/t	2/*q*t	3crom/*p*i	-	19,20
*C. vanderi*	Preto River/SP	54	108	54m/sm	6/*q*t	-	-	-	-	2
*C. voga*	Bolacha Stream/RS	54	108	54m/sm	6/*q*t	-	-	-	-	2
	Paraná River/AR	54	108	54m/sm	*q*t	pc/t/i	-	-	-	11
	Saco da Alemoa River/RS	54	108	54m/sm+B	5/*q*t	pc/t	5/*q*t	-	-	19,20
	Capivara Stream/RS	54	108	54m/sm+B	5/*q*t	pc/t	5/*q*t	-	-	19,20
	Gasômetro/RS	54	108	54m/sm+B	5/*q*t	pc/t	5/*q*t	-	-	19,20
	Barros Lagoon/RS	54	108	54m/sm+B	5/*q*t	pc/t	5/*q*t	2crom/*p*i	-	19,20
	Quadros Lagoon/RS	54	108	54m/sm+B	5/*q*t	pc/t	5/*q*t	-	-	19,20
*C. saladensis*	A.E.S. UFRGS Dam/RS	54	108	54m/sm+B	8/*q*t	pc/t	8m/*q*t	2crom/*p*i	-	19,20
*Potamorhina*										
*P. altamazonica*	Negro and Solimões River/AM	102	106	2m+2sm+98a	5a/*q*t	pc/t/i	5a/*q*t	41a/*q*i	t	4,21
*P. latior*	Negro and Solimões River/AM	56	112	52m+2sm+2st	25m/*q*t	pc/t/i	25m/*q*t	4m/*p*t	t/ITS 18 pairs	4,21
*P. pristigaster*	Negro and Solimões River/AM	54	108	42m+12sm	25sm/*p*t	pc	-	-	-	4
	Negro and Solimões River/AM	54	108	44m+10sm	5m/*q*t	pc/t	5m/*q*t	4m/*p*t	t/ITS 1 pair	21
*P. squamoralevis*	Paraná River/AR	102	116	14m/sm+88a	*qt*	pc	-	-	-	11
*Psectrogaster*										
*P. amazonica*	Araguaia River/MT	54	108	44m+10sm	17m/*p*t	-	-	-	-	16
*P. curviventris*	Miranda River/MS	54	108	42m+12sm	20m/*p*t	pc	-	-	-	7
	Paraná River/AR	54	108	54m/sm	*qi*	pc/t	-	-	-	11
*P. falcata*	Araguaia River/PA	54	108	40m+14sm	13m/*p*t	pc/t	13m/*p*t	24sm/*p*i	t/ITS 15 pairs	22
*P. rutiloides*	Negro and Solimões River/AM	54	108	42m+12sm	9m/*q*t	-	-	-	-	3
	Catalão Lake/AM	54	108	46m+8sm	16m/*p*t	pc/t/i	16m/*p*t	5m/*p*t 22sm/*q*i	t/ITS 18 pairs	22
*Steindachnerina*										
*S. amazonica*	Araguaia River/GO	54	108	42m+12sm	2m23sm/*q*t	pc/t	-	-	-	16
*S. biornata*	Forquetinha River/RS	54	108	54m/sm+B	3m/*q*t	pc/t	4crom/*q*t	-	-	19,20
*S. brevipinna*	Miranda River/MS	54	108	46m+6sm	17m/*p*t	c/t	-	-	-	7
	Paraná River/AR	54	108	54m/sm	15m/*q*t	pc/i/t	-	-	-	11
*S. conspersa*	Paraguai River/MS	54	108	54m/sm	2m/*q*i	-	-	-	-	2
	Paraná River/AR	54	108	54m/sm	2m/*q*t	pc/t/i	-	-	-	11
*S. elegans*	São Francisco River/SP	54	108	54m/sm	25/*p*t	-	-	-	-	2
*S. gracilis*	Araguaia River/MT	54	108	38m+16sm	4crom/*q*t	-	-	-	-	16
*S.* cf *guentheri*	São Francisco River/AC	54	108	54m/sm	24/*p*t	pc/i/t	-	-	-	9
*S. insculpta*	Mogi-Guaçu River/SP	54	108	54m/sm	25/*p*t	-	-	-	-	2
	Passa-Cinco River/SP	54	108	54m/sm	25/*p*t	-	-	-	-	2
	Paranapanema River/SP	54	108	54m/sm+B	7/*q*t	pc/t	7/*q*t	2/*p*i	-	5,12,14,17
	Reserve Jurumirim/SP	54	108	54m/sm+B	-	pc	-	-	-	5
	Tietê River/SP	54	108	54m/sm	7/*q*t	pc/t	7/*q*t	2/*p*i	-	12,14,17
	Três Bocas Stream/PR	54	108	54m/sm+B	7/*q*t	pc/t	7/*q*t	-	-	13,15
	Taquiri River/PR	54	108	54m/sm	7/*q*t	pc/t	7/*q*t	-	-	13,15
	Tibagi Rio/PR	54	108	54m/sm	7/*q*t	pc/t	7/*q*t	-	-	13,15
	Água da Floresta River/PR	54	108	54m/sm	7/*q*t	pc/t	7/*q*t	-	-	13,15
	Emas Waterfall/SP	54	108	50m+4sm	22m/*p*t	pc/t	-	-	-	16
	Água dos Patos River/SP	54	108	54m/sm+B	12/*p*t	pc/t	12/*p*t	2crom/*p*i	-	19, 20
	Três Bocas Stream/PR	54	108	54m/sm+B	12/*p*t	pc/t	12/*p*t	2crom/*p*i	-	19, 20
	Pavão Stream/PR	54	108	54m/sm+B	12/*p*t	pc/t	12/*p*t	-	-	19, 20
	Jacutinga River/PR	54	108	54m/sm+B	12/*p*t	pc/t	12/*p*t	-	-	19, 20
*S. leucisca*	Negro and Solimões River/AM	54	108	48m+6sm	15m/*p*t	-	-	-	-	3

1- Venere and Galetti (1985); 2- Venere and Galetti (1989); 3- Feldberg et al. (1992); 4- Feldberg *et al*. (1993); 5- Oliveira and Foresti (1993); 6- Martins et al. (1996); 7- Navarrete and Júlio Jr (1997); 8- Venere *et al*. (1999); 9- Carvalho et al. (2001); 10- Fenocchio et al. (2003); 11- Brassesco et al. (2004); 12- De Rosa et al. (2006); 13- Gravena et al. (2007); 14- De Rosa *et al*. (2007); 15- Teribele et al. (2008); 16- Venere *et al*. (2008); 17- De Rosa *et al*. (2008); 18- Oliveira (2010); 19- Sampaio et al. (2011); 20- Sampaio *et al*. (2016); 21- Pinheiro et al. (2016); 22- Present study.

The present study aims to investigate the chromosomal composition and structure of the karyotypes of six Amazonian Curimatidae species. The results were compared with the data available in the literature to infer the hypothetical chromosomal rearrangements involved in the chromosomal evolution process.

A total of 52 individuals from six species of the Curimatidae family were cytogenetically analyzed (Table 2). The fishes were collected under authorization from the Instituto Chico Mendes de Conservação da Biodiversidade (ICMBio, SISBIO - 28095-1). All procedures followed the guidelines of the Ethics Committee for Experimental Use of Animals of the National Institute of Amazonian Research (004/2018-CEUA/INPA), and the specimens were deposited in the INPA Ichthyology Collection (INPA-ICT 059622 - INPA-ICT 059627).

**Table 2-:** Cytogenetic data of fish species from Curimatidae family analyzed in this study. M= male; F= female; ?= Unknown sex; 2n= diploid number; FN= fundamental number; Ag-NOR= nucleolar organizer regions; rDNA= ribosomal DNA; m= metacentric; sm= submacentric; p= short arm; q= long arm; t= terminal; i= interstitial.

Species	Sex			Locality / Coordinates	2n	FN	Karyotype structure	Ag-NOR Pair / Position	18S rDNA Pair / Position	5S rDNA Pair / Position
	M	F	?							
*Curimata inornata*	-	-	8	Araguaia river, PA 5°25’33.59”S 48°28’30.37”W	54	108	38m + 16sm	20sm/*p*t	20sm/*p*t	9m/*p*i
*Curimata vittata*	-	2	-	Catalão lake, AM 3°09’42.2”S 59°54’54.7”W	54	108	38m + 16sm	20sm21sm/*q*t	20sm21sm/*q*t	25sm/*p*i
*Curimatella dorsalis*	-	2	-	Araguaia river, PA 5°25’33.59”S 48°28’30.37”W	54	108	44m + 10sm	2m/*q*t	2m/*q*t	2m/*q*i
*Curimatella meyeri*	4	13	-	Catalão lake, AM 3°09’42.2”S 59°54’54.7”W	54	108	46m + 8sm	9m/*q*t	7m/*p*t 9m/*q*t	26sm/*p*i
*Psectrogaster falcata*	-	-	3	Araguaia river, PA 5°25’33.59”S 48°28’30.37”W	54	108	40m + 14sm	13m/*p*t	13m *p*t	24sm/*p*i
*Psectrogaster rutiloides*	10	10	-	Catalão lake, AM 3°09’42.2”S 59°54’54.7”W	54	108	46m + 8sm	16m/*p*t	16m/*p*t	5m/*p*t22m/*q*i

We followed the protocol described by Gold et al. (1990) to obtain the mitotic chromosomal preparations. Constitutive heterochromatin (CH) was detected according to Sumner (1972), with modifications where the staining was performed with a solution containing 0.5 μL of propidium iodide in 20 μL of Vectashield®, according to Lui et al. (2012). Active nucleolar organizer regions (NORs) were identified using the silver staining method, according to Howell and Black (1980).

For molecular cytogenetic analyses, genomic DNA was extracted from muscle, according to Sambrook et al. (1989). Ribosomal DNA (rDNA) 18S, 5S, and telomeric probes were amplified by Polymerase Chain Reaction (PCR) using the following primers: 18Sf (5’-CCGCTTTGGTGACTCTTGAT-3’) and 18Sr (5’-CCGAGGACCTCACTAAACCA-3’) (Gross et al., 2010), 5Sf (5’ -TAC GCC CGA TCT CGT CCG ATC) and 5Sr (5 ‘-CAGGCT GGT ATG GCC GTA AGC- 3’) (Martins and Galetti Jr., 1999), (TTAGGG) 5 and (CCCTAA) 5 (Ijdo et al., 1991). Probes were labeled using nick-translation with biotin-14-dATP (Biotin Nick Translation Mix; Invitrogen) for 5S rDNA and digoxigenin-11-dUTP (Dig-Nick Translation Mix; Roche) for 18S rDNA and telomere, following the recommendations of the manufacturer.

FISH followed Pinkel et al. (1986), with modifications. The slides with chromosome preparations were denatured in 70% formamide/2x SSC at 70 °C, pH 7, and dehydrated in 100% ethanol. Then, 20 μL of hybridization mix (100 ng of each probe, 100% formamide, 20x SSC buffer, and 10% dextran sulfate) were placed on each slide, being hybridized at 37 °C for 24 h in a humid chamber, containing distilled water. Chromosomes were counterstained with DAPI (1.2 μg mL) in an antifade solution (Vector, Burlingame, CA, EUA).

At least 30 metaphase spreads of each individual were analyzed to confirm the diploid number and karyotype structure. The chromosomes were classified as metacentric (m) and submetacentric (sm) (Levan et al., 1964).

The six species analyzed presented 2n=54 and FN=108 (Fundamental number) (Figure 1, Table 2), it is highlighted that the karyotype of *Psectrogaster falcata* is presented here for the first time. CH was observed in pericentromeric blocks in all chromosomes of the six species, except in pairs 5 and 18 of *P. falcata*. Furthermore, additional blocks located in the terminal portions of several chromosomes of the six species were also observed. *C. meyeri* showed interstitial blocks in the long arms of pair 5; and pairs 2, 19, and 21 in *P. rutiloides* (Figure 1).

**Figure 1.**
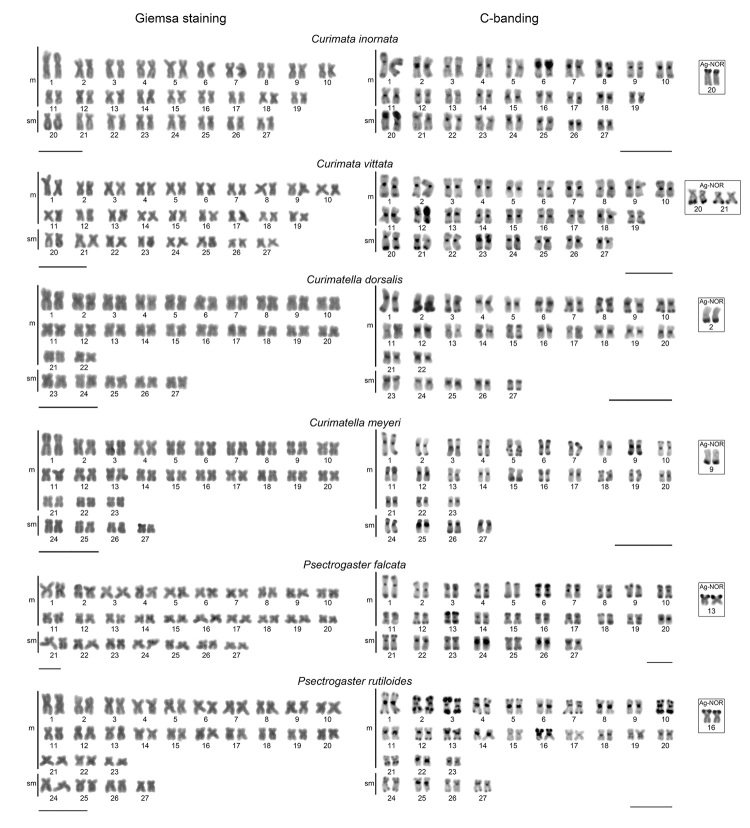
Karyotypes of the species of the Curimatidae family analyzed in conventional Giemsa stain (left), C banding (right) and nucleolar organizer regions (NOR, box). Scale bar=10μm.

Five species presented NOR in only one chromosome pair in the terminal portion of the short arms: *C. inornata*, *P. falcata*, and *P. rutiloides* (pairs 20, 13, and 16, respectively), and in the end of the long arms in *C. dorsalis* and *C. meyeri* (pairs 2 and 9, respectively). *C. vittata* exhibited NORs in two chromosome pairs (multiple NORs) in the terminal portion of the long arms (pairs 20 and 21). The six species showed the NORs colocalized with heterochromatic blocks (Figure 1, box Ag-NOR).

The rDNA mapping corroborates the NORs in all the species studied, including an additional site observed in the end of the short arm of pair 7 in *C. meyeri*, which is also colocalized to the constitutive heterochromatin (Figure 2, 18S). The 5S rDNA sequences mapping revealed a species-specific pair with interstitial signals: pair 9 in *C. inornata*, pair 25 in *C.* vittata, pair 2 in *C. dorsalis*, pair 26 in *C. meyeri*, and pair 24 in *P. falcata*. *P. rutiloides* presented 5S sites in two pairs: pair 5 in the terminal portion of the short arm and interstitial in pair 22. *C. dorsalis* showed synteny of 5S and 18S (Figure 2). Telomeric sequences (TTAGGG)_n_ were located in the terminal region of all chromosomes of the six species. Additionally, interstitial telomeric sequences (ITSs) were observed in several chromosomes of *Curimatella* and *Psectrogaster* species, with some conspicuous blocks (Figure 2).

**Figure 2.**
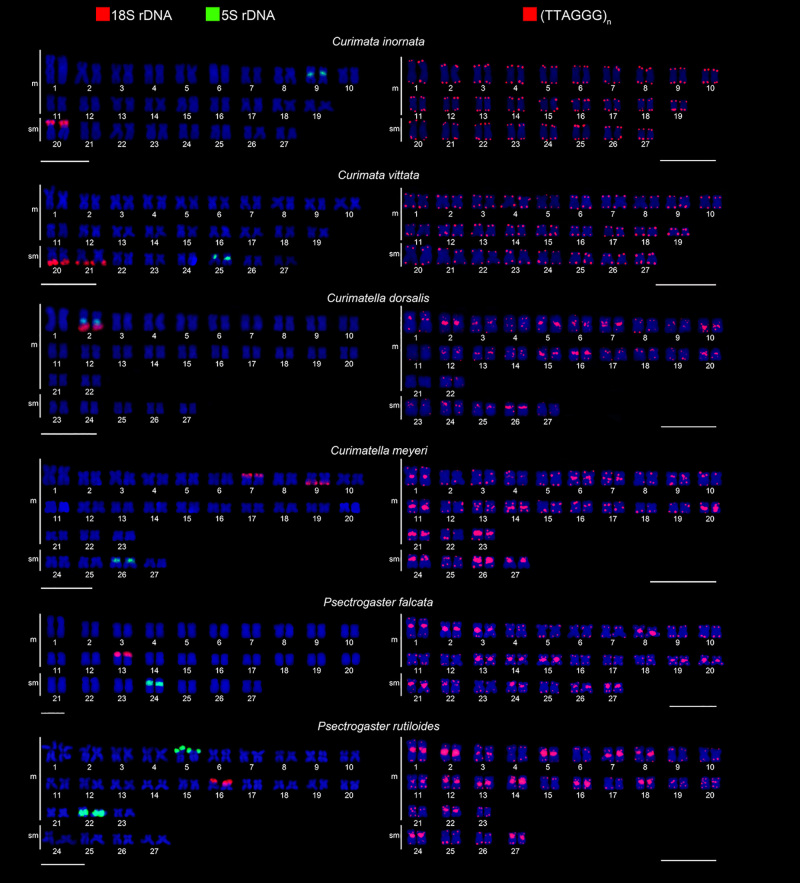
Karyotypes of the species of the Curimatidae family analyzed with molecular chromosomal markers. Double FISH with 18S (red) and 5S (green) rDNA probes (left), and probes with telomeric sequences (TTAGGG)n (red) (right). Scale bar = 10μm.

Chromosomal evolution of the family Curimatidae was defined as being highly conservative chromosome morphology and diploid number: 2n=54 m-sm, FN=108 for the majority of the species (Table 1). These traits, considered plesiomorphic for the family, were also evidenced in the species analyzed here in. According to Oliveira et al. (1988) and De Oliveira et al. (2009), this conservative chromosomal structure may be related to the ecological characteristics of these fishes, that is, high vagility and large shoal formation, allowing high rates of gene flow and genetic diversity (Landínez-García and Marquez, 2018). However, this apparent conservation is revealed when other cytogenetic markers, such as repetitive DNA sequences (e.g., ribosomal and telomeric) are applied.

Curimatids, in general, have a large amount of HC, and in *Psectrogaster* species for example, pericentromeric and terminal blocks were observed in several chromosome pairs (Figure 1). Beyond that, large heterochromatic blocks are often coincident or adjacent to the NORs, with interspecific and interpopulation differences, both in the number of *loci* (single or multiple NORs) and in the chromosomal location/position in the karyotype (Table 1), as seen in the present study as well as in previous studies (Feldberg et al., 1992; Navarrete and Júlio-Júnior, 1997; Brassesco et al., 2004; Venere et al., 2008). These differences may be related to the repetitive and highly transcribed structure of rDNA, where the number of copies might vary owing to rearrangements of the chromosomal microstructure, such as duplications, translocations and/or inversions (Symonová et al., 2013; Goffová and Fajkus, 2021).

The mapping of the 18S rDNA sequence confirmed Ag-NOR in all species with an additional site in *C. meyeri*, similar situation also reported by Sampaio et al. (2016) in *Steindachnerina biornata*. This additional site might be related to the lack of transcriptional activity, which depends on cell activity (Rosa et al., 2012), or simply associated with the presence of pseudogenic rDNA variants (Gong et al., 2021).

The 5S rDNA localization in interstitial region, ranging from two to four chromosomes, is a pattern found in most curimatids corroborated in the present study. However, markings in terminal chromosomal regions have also been reported in this family (Pinheiro et al., 2016; present study), again evidencing the occurrence of non-Robertsonian rearrangements in chromosome microstructure of these species.

The location of 18S and 5S rDNA in different chromosome pairs is a trait found in all curimatid species (Table 1). Interestingly, *Curimatella dorsalis* seems to be the first case to show synteny between these rDNAs in curimatids, which may have arisen independently during non-Robertsonian rearrangements (Symonová et al., 2013), demonstrating the dynamic nature of the 18S and 5S rDNA sites, prone to recombination events. Synteny between 18S and 5S rDNA is an atypical situation, including for the superfamily Anostomoidea (Anostomidae, Chilodontidae, Prochilodontidae and Curimatidae), which has been reported only in lineages derived of the Anostomidae (De Barros et al., 2017; Dulz et al., 2019), Prochilodontidae (Vicari et al., 2006; Terencio et al., 2012; Voltolin et al., 2013) and Curimatidae families (present study).

Chromosome mapping of telomeric sequences revealed a high degree of chromosome structure variation in *Curimatella* and *Psectrogaster* species, presenting ITSs in several chromosome pairs. ITS has been observed in several vertebrate species and is classified into short ITS (s-ITS) and heterochromatic ITS (Het-ITS) (Bolzán, 2017). In the case of the curimatids here analyzed, we classify the ITSs as Het-ITSs, since the signals are colocalized with heterochromatic blocks.

Many authors relate the presence of Het-ITSs to ancestral chromosomal fusion events and are generally associated with a reduction in diploid number (Meyne et al., 1990; Rosa et al., 2012; Schneider et al., 2013; Sember et al., 2015). Similarly, there are reports of Het-ITSs in species that present the conserved karyotype (Metcalfe et al., 2004; Di-Nizo et al., 2020), as observed in the present study, considering that 2n=54 is the ancestral diploid number for the whole superfamily Anostomoidea.

Thus, the appearance of these Het-ITSs may be related to other mechanisms, such as (1) occurrence of pericentric inversions or translocations with the insertion of s-ITSs, followed by amplification of these regions and subsequent heterochromatinization; (2) transpositions, mediated by transposable elements, which are internally reinserted into the chromosomes and undergo an amplification process; and, (3) telomeric sequences (TTAGGG)_n_ would constitute the main repetitive motif of centromeric DNA, as observed in amphibians and marsupials (Meyne et al., 1990; Paço et al., 2012; Bolzán, 2017; Clemente et al., 2020).

Regardless of the mechanism that gave rise to Het-ITSs in the curimatids here analyzed, these sequences are an important component of the karyotype diversification. As observed in another genus of Curimatidae, in *Potamorhina* ITSs are involved in multiple chromosomal fissions in the ancestor of the species *P. latior* (2n=56, 18 pairs with ITS), *P. altamazonica* (2n=102), and *P. squamoralevis* (2n=102) (Pinheiro et al., 2016), as suggested in molecular phylogeny of Dorini et al. (2020). Thus, the Het-ITSs present in *Curimatella* and *Psectrogaster* can signal the presence of “hot spots” for the occurrence of recombination, which according to Bolzán (2017), can lead to new karyotypes and even new species.

Thus, despite the conservative diploid number for most species of the Curimatidae (2n=54), our data highlights a high level of variation in repetitive DNA sequences among species, suggesting that additional integrative analyzes, involving the mapping of other repetitive sequences classes as well as investigation in other species/populations of curimatids, will produce a more complete picture of the chromosomal evolution of this family.
